# Topic modeling and evolutionary trends of China’s language policy: A LDA-ARIMA approach

**DOI:** 10.1371/journal.pone.0324644

**Published:** 2025-05-28

**Authors:** Tianxin Li, Xigang Ke, Hui Shi

**Affiliations:** 1 Department of Literature, Shaanxi Normal University, Xi’an, Shaanxi, China; 2 Department of Literature, Nanjing Normal University, Nanjing, Jiangsu, China.; National Chengchi University, TAIWAN

## Abstract

**Background:**

Language policy serves as an essential tool for governments to guide and regulate language development. However, China’s current language policy faces challenges like outdated analytical methods, inefficiencies caused by policy misalignment, and the absence of predictive frameworks. This study provides a comprehensive overview of China’s language policy by identifying key topics and predicting future trends.

**Methods:**

We employ the Latent Dirichlet Allocation topic model and Autoregressive Integrated Moving Average model systematically analyze and predict the evolution of China’s language policy. By gathering a large-scale textual data of 1,420 policy texts from 2001–2023 on official websites, we achieve both topic extraction and evolution prediction.

**Results:**

This study reveals that: (1) Language life, language education, and language resources have high popularity indexes, and language education and language planning exhibit high expected values. (2) The theme intensity of most topics has been a significant upward trend since 2014, with significant fluctuations during T1-T2. (3) From 2001 to 2023, the actual and fitted values show an overall positive trend. In 2024–2028, the predicted value of language resources stabilizes after a brief decline in 2024, while other topics show upward trends.

**Conclusions:**

This study extracts 1,420 policy texts from official websites and outlines the following findings: (1) Language policies focus on maintaining a harmonious linguistic environment, addressing educational inequality, and protecting language resources. (2) Since 2014, most topics have exhibited fluctuating yet sustained growth trend, particularly in language education and research. (3) Except for language resources, the predicted values of the remaining six topics will show a growing trend from 2024 to 2028. Based on these findings, we propose policy recommendations such as strengthening language research, developing a multilingual education system, and optimizing language resource management.

## Introduction

Language policy is defined as the laws, regulations, rules, and measures established by human social groups to govern speech communication based on their position and viewpoint on a certain language [1,2]. In China, the language service industry generated 55.45 billion yuan in 2021, representing approximately 16.66% of the global market [3]. The effectiveness of language policy plays a significant role in shaping economic restructuring, driving technological innovation, and maintaining cultural sovereignty [4,5]. However, the rapid digitization of language technologies has exposed a significant gap: China’s current language policy frameworks, which primarily rely on retrospective qualitative evaluations [6–8], are insufficiently responsive to the real-time challenges posed by innovation-driven development strategies [9].

Language policies face three key challenges. First, traditional policy analysis methods like expert reviews and manual coding, are increasingly inadequate due to subjective biases, slow feedback, and limited ability to handle large amounts of multilingual data from digital platforms [10,11]. Second, despite the rapid growth of China’s language industry, policy misalignment has led to inefficiencies [12], with overinvestment in machine translation while underfunding of minority language preservation [13,14]. Third, as AI-driven language technologies reshape global labor markets, the absence of timely policy adjustments has contributed to workforce disruptions and widening linguistic inequalities in several countries [15,16]. China currently lacks a predictive framework capable of anticipating and mitigating these risks.

Previous research has inadequately addressed these issues. Existing studies on language policy phases remain descriptive, failing to account for the real-time interactions between policy texts and socio-technological outcomes. Although computational methods like topic modeling and time series analysis have been widely applied in fields such as energy policy [17], monetary policy [18], and healthcare policy [19], they have yet to address the complexities of language governance, such as dialect diversity and policy terminology’s ideological impact. This gap limits policymakers’ ability to assess interventions like AI ethics guidelines or regional language revitalization campaigns.

To bridge this gap, we propose a machine learning framework integrating Latent Dirichlet Allocation (LDA) [20,21] and Autoregressive Integrated Moving Average (ARIMA) models [22]. The main contributions are as follows: (1) Introducing a machine learning method for evaluating language policy that shifts from text analysis to policy effect assessment, offering new methods and perspectives for precise policy identification. (2) Employing the LDA model to systematically and accurately evaluate key topics and their relationships, enhancing existing literature. (3) Applying ARIMA model to enable real-time monitoring and feedback on language policy implementation, aiding policymakers in making prompt adjustments and enhancements to ensure policy efficacy and flexibility.

## Literature review

### Progress in language policy and text mining

Language policy plays a crucial role in managing national society, resolving political conflicts and centralizing political resources [23–25]. In China, early language policy efforts focused on macro-level planning [26], including implementing comprehensive language and writing reforms, such as the organization and simplification of Chinese characters and the widespread promotion of Mandarin in 1949 [27]. In the 21st century, language policy has gradually shifted from a macro to a micro focus, addressing linguistic diversity and social needs [28]. Most existing studies on language policy concentrate on policy implementation and evaluation [29–31]. Research on policy implementation explore the evolution of language policy [32], social network analysis [33], discourse analysis [34], and cross-national comparisons [35]. Policy evaluation studies, on the other hand, focus on language return rates [36], the socio-economic effects of language policy [37], cost-benefit analysis [38], and future prospects [39].

Despite these advancements, much research remains largely qualitative and descriptive, relying on case studies and expert analysis. Moreover, large-scale, data-driven methods are underutilized for dynamic policy impact assessment. Text mining, a computational technique for extracting meaningful patterns from unstructured texts, has gained increasing attention in policy research [40]. Scholars have shown great interest in large-scale policy documents, including government policy texts, legal records, policy news and media data [41–43]. Common text mining techniques such as text clustering, text classification, and feature extraction have been widely used and validated [44–46]. Policy text mining finds applications in text information retrieval, sentiment analysis, theme evolution analysis, and performance evaluation [47–49]. Natural language processing (NLP) methods based on big data can transform policy texts into structured data, facilitating the construction of specialized objects such as semantics or sentiment [50,51].

However, the application of text mining in language policy research remains underdeveloped. While its successful implementation in energy regulation, economic policy, and healthcare policy [52–54], language policy presents unique challenges, including dialect diversity, ideological nuances, and regional variations. Some studies focus on short-term policies, limiting their ability to predict shifts and assess long-term impacts [55]. Furthermore, many studies rely on qualitative methods [56], which may lead to a lack of objectivity. Therefore, there is a need to incorporate quantitative methods for more precise analysis. Utilizing NLP techniques based on big data can offer a more systematic, scalable, and objective evaluation of policy effectiveness [57].

### Topic modeling and evolutionary trends

Topic modeling extracts key content from texts, uncovers hidden policy insights, and identifies topic relationships [58,59]. This process aids in enabling individuals to promptly comprehend the trends of language policy development, providing the basis for in-depth analysis of language issues and strengthening the role of policy on social and economic development.

At present, there are a large number of academic research, primarily focusing on topic modeling and visualization [60–62]. Scholars have concentrated on the current state of topic modeling, such as the work of Blei, an American academic, who utilized the LDA topic model to reveal hidden subject information within extensive texts or corpora [20]. Furthermore, some researchers have analyzed the temporal dynamics in the socio-political landscapes during presidential elections, analyzing political texts to explore the multi-faceted nature of public policy [63]. Other scholars have expanded on evolutionary analysis by integrating the weighted Jacobian matrix with LDA analysis to illustrate the trend of topic change [64].

As research has progressed, several studies have sought to better capture topic evolution over time, though challenges remain. In response, scholars have made notable attempts to address this gap. For instance, after constructing theme models, some scholars have utilized sentiment classification models to categorize the derived topics [65]. Several studies employed the ARIMA model to forecast the derived topics [66]. Others have combined time series analysis with support vector machine model, generating time series data through LDA topic modeling and using support vector machine model for trend analysis [67,68]. Furthermore, combining topic modeling with evolutionary trends presents challenges. One issue is that topics evolution is often nonlinear, making it difficult for traditional time series models to capture their shifts. In addition, the integration of qualitative thematic content with quantitative forecasting introduces complexity, highlighting the need for more advanced approaches that consider both semantic features and temporal dynamics.

In summary, most existing studies focus on the visualization analysis of topic modeling. While some studies have started to explore evolutionary trends, the predominant approach involves constructing time series models and interpreting these trends manually. To date, few research has combined topic evolution and time series analysis on language policy. Given the strengths of LDA model, such as its strong dimensionality reduction capabilities [69], robust foundation in probability theory [70], and scalability [71], along with the ARIMA model’s accurate short-term predictive capabilities for analyzing time-dependent data, this paper aims to bridge this gap. Specifically, we collect China’s language policy texts from official websites from 2001 to 2023. The research employs the LDA theme model to identify key topics and constructs an ARIMA model to predict the theme intensity of language policy from 2024 to 2028. This integrated approach synthesizes internal semantic features and external theme intensity to analyze theme evolution trends and provide predictive assessments.

## Methods

### Dataset

The study obtained language policy texts from the official website of the Chinese government (https://www.gov.cn/) using the keyword “语言” (language) and from the “国内语情” (Domestic Language Situation) of the General Office of the National Language Commission Research Planning Committee (http://www.ywky.edu.cn/) from January 2001 to October 2023. Both sources are authoritative Chinese government websites. After removing incomplete, redundant, or irrelevant texts, 1,420 policy texts were selected as the main research sample, ensuring the dataset’s integrity and quality.

### Data preprocessing

Thematic analysis typically requires data preprocessing to refine the data, which includes three key tasks:

(1) Data cleaning. We remove extraneous content like HTML tags, special characters, noise, null values and duplicate entries from the policy text to ensure the data’s consistency and suitability for topic modeling. In addition, we convert relevant English terms to lowercase, while keeping key numerical values for context.
(2) Text Segmentation. Because Chinese lacks explicit word boundaries, we employ Python’s Jieba tokenizer for segmentation, ensuring accurate identification of multi-character policy terms.
(3) Punctuation and stopword removal. To enhance the quality of text mining, we remove punctuation marks, such as period (“.”), comma (“,”), question mark (“?”), exclamation point (“!”), and special characters including ampersand (“&”) and slash (“/”). In addition, we eliminate stopwords—such as “和”, “是” and “的”—that do not contribute to semantic meaning. The stopword list is sourced from a public website (https://countwordsfree.com/stopwords).

### Utilizing latent dirichlet allocation for topic identification

This study employs LDA topic modeling for thematic analysis of language policy texts. LDA, an unsupervised machine learning method, uses generative probabilistic techniques to deduce word distributions that characterize various topics [72]. This approach allows for the examination of policy texts with diverse topics and complex lexical features, converting textual information into a digital format. The fundamental equation is denoted as (1):


$$\mathrm{\backslash [}p\left(\theta ,z,w|\alpha ,\beta \right)=p\left(\theta |\alpha \right)\prod_{n=1}^{N}p\left(z_{n}|\theta \right)p\left(w_{n}|z_{n},\beta \right)\;\;\;\;\;\;\mathrm{\backslash ]}$$ (1)

Where θ represents the topic distribution for a document, while z and w denote the sequence of topic assignments and the sequence of words in the document. The hyperparameters α and β govern the Dirichlet prior distributions, p(θ|α) models the probability of the topic distribution conditioned on the Dirichlet prior α. For each word in the document, $p\left(z_{n}|\theta \right)\;$ represents the probability of assigning topic $z_{n}$ to word $w_{n}$, and $p\left(w_{n}|z_{n},\beta \right)\;\;$ denotes the probability of generating word $w_{n}$ given its assigned topic $z_{n}$, and the word distribution parameter β. The product notation ∏ accounts for all words n in the document.

We preprocess the data to create a corpus for LDA analysis, following these steps: (1) We vectorize policy texts using the Bag-of-Words (BOW) model and apply Term Frequency-Inverse Document Frequency (TF-IDF) weighting to refine word importance. (2) We train an initial LDA topic model while determining the optimal number of topics by minimizing perplexity and maximizing coherence, ensuring topic quality and interpretability [73,74]. (3) Using the optimal number of topics, we train the final LDA model with the selected hyperparameters, producing topic-word and document-topic distributions. (4) We calculate the theme intensity over time for each topic to analyze the evolving trends in language policy.

### Evolutionary trends based on autoregressive integrated moving average model

ARIMA, a widely used statistical method for time series forecasting, which involves curve fitting and parameter estimation to develop a mathematical model for future predictions. It is applied in various fields, including economics, finance, hydrological forecasting, and aerospace [75–78]. We apply the ARIMA model to forecast theme intensity, represented as ARIMA (p, d, q), where p represents the autoregressive term, d signifies the difference order, and q denotes the number of moving average terms. The calculation is detailed in Equation (2).


$$\Delta^{d}y_{t}=\alpha \sum_{i=1}^{p}\gamma_{i}\Delta^{d}y_{t-i}+\beta \sum_{i=1}^{q}\theta_{i}\varepsilon_{t-i}+\varepsilon_{t}+\mu$$ (2)

**Fig 1:**
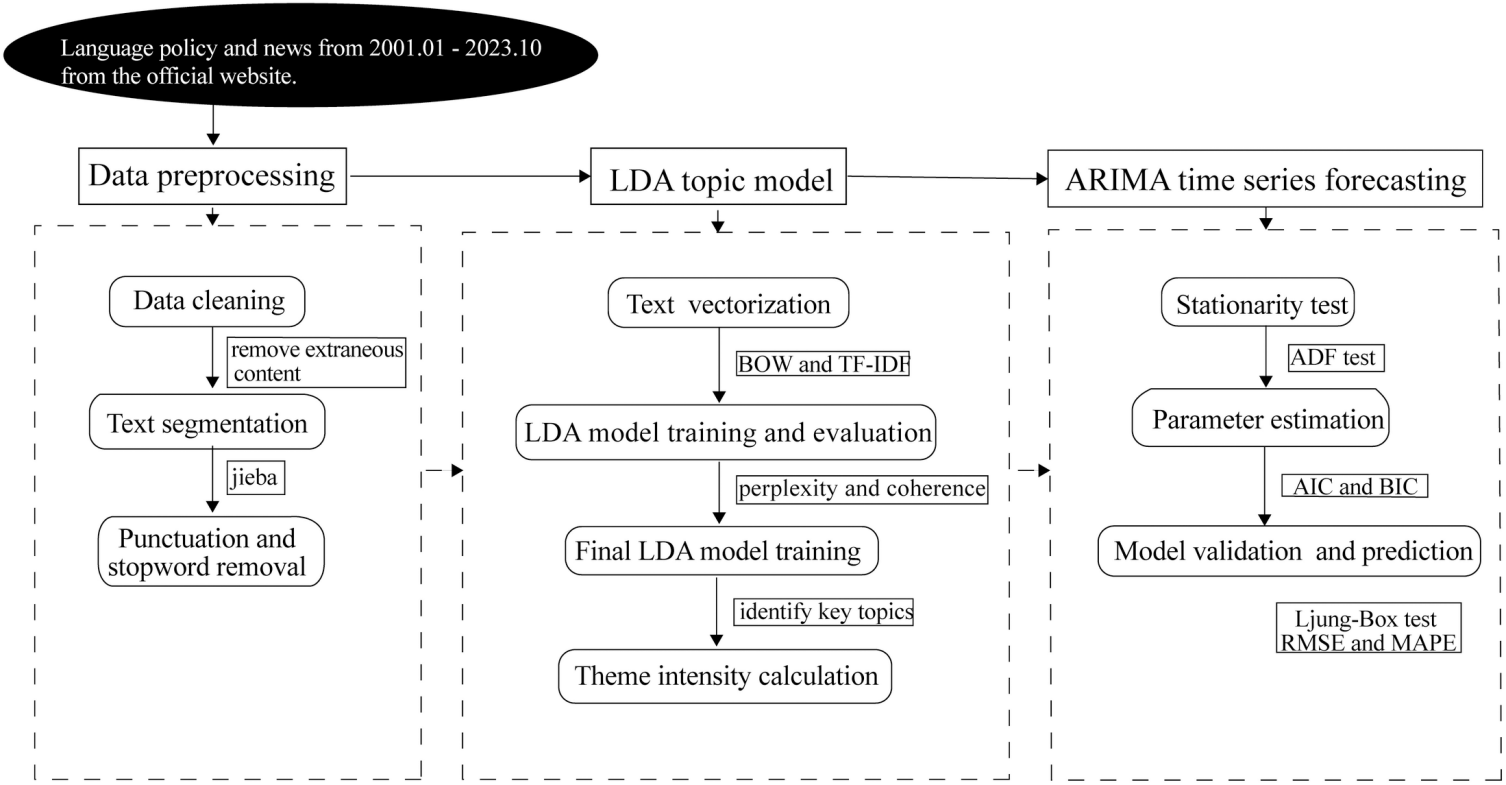
The flowchart of the proposed method.

Where $\Delta^{d}y_{t}$ is the *d*-th order differenced value of the original time series $y_{t}$, $\gamma_{i}$ is the autocorrelation coefficient, $\theta_{i}$ is the moving average coefficient, $\varepsilon_{t}$ is the error, μ is the constant term, and α and β are the coefficients. The process of using the ARIMA model to analyze theme intensity evolution involves three key steps:

(1) Stationarity test. We assess stationarity using the Augmented Dickey-Fuller (ADF) test, where the null hypothesis assumes a unit root (non-stationarity). If the ADF statistic is higher than the 5% critical value, the series is non-stationary, and first-order differencing is applied. This process is repeated until stationarity is confirmed.
(2) Parameter estimation. To estimate model parameters, we first examine the Autocorrelation Function (ACF) and Partial Autocorrelation Function (PACF), though subjective factors may influence interpretation. Following the Box-Jenkins methodology, we complement this approach with the Akaike Information Criterion (AIC) and Bayesian Information Criterion (BIC) [79,80]. To balance forecasting accuracy and model complexity, we explore the autoregressive (AR) and moving average (MA) orders over the ranges 0 ≤ p ≤ 2, and 0 ≤ q ≤ 2. The optimal parameter is selected based on $\frac{\left(AIC+BIC\right)\;}{2}$ minimum [81].
(3) Model validation and prediction. We validate model adequacy by applying the Ljung-Box test to residuals [82], where p > 0.05 indicates white noise. Predictive performance is assessed using out-of-sample rolling forecasts, measured by Root Mean Squared Error (RMSE) and Mean Absolute Percentage Error (MAPE). The final ARIMA(p,d,q) model is then used to forecast trends for the next five years. The entire process is shown in Fig 1.

## Results

### Determining the optimal number of topics

**Fig 2:**
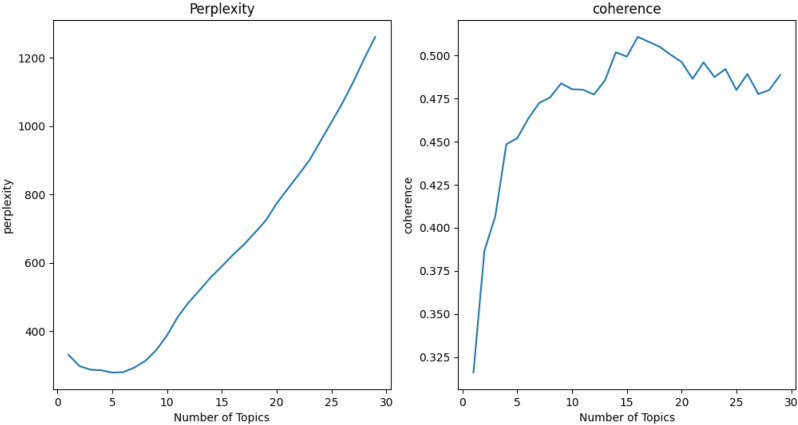
Perplexity and coherence of LDA models with different values of K^a^. ^a^ The figure examines the range of topics K from 0 to 30, utilizes a step size of 5 for LDA topic extraction, and evaluates perplexity and coherence on the test dataset.

To determine the optimal number of topics, researchers have explored several quantitative and qualitative methods such as perplexity, coherence [83,84]. This study integrated perplexity and coherence to identify the optimal number of topics. Fig 2 demonstrated that LDA exhibited lower perplexity and higher coherence when K = 7, suggesting that the optimal number of topics was 7.

### Policy text keywords analysis

**Fig 3:**
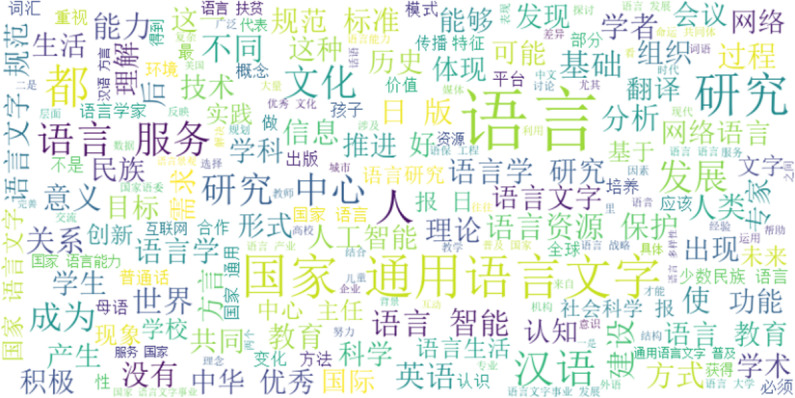
The high-frequency word cloud of China’s language policy.

The word cloud in Fig 3 illustrated high-frequency keywords central to language policy: language, common language, research, country, development, culture, education, construction, and service. These keywords collectively outlined the core aspects of language policy, encompassing research, education, and services. In research, language policies addressed language resources, cognition, and life. In education, policies focused on language proficiency, schools, students, common language, and dialects. Regarding services, they emphasized the integration of translation services for English and Chinese, as well as AI technologies.

### Topic modeling results

#### Topic identification and popularity analysis.

To identify research directions and hotspots, Table 1 displayed the parameters related to the probability distribution, including popularity index, expected value, standard deviation and dispersion coefficient. We had the following findings: (1) Topic 1 had the highest popularity index of 0.318, followed by Topic 2 and 3. However, Topic 6 and 7 had the lowest popularity index of 0.045, indicating their marginal status. (2) Most topics exhibited relatively high expected values, except for Topic 7. Notably, Topic 2 and 6 had the highest expected values of 0.154, warranting further exploration of their unique attributes. (3) Topics 2, 3 and 6 had higher dispersion coefficients, indicating that language education, language resources and language planning exhibited broader distributions and potentially greater diversity.

**Table 1:** Statistical parameters of China’s language policy.

	Topics	Popularity index	Expected value	Standard deviation	Dispersion coefficient	Rank
1	Language life	0.318	0.153	0.002	0.015	1
2	Language education	0.227	0.154	0.009	0.056	2
3	Language resources	0.182	0.152	0.004	0.029	3
4	Language protection	0.091	0.151	0.001	0.007	4
5	Language research	0.091	0.153	0.001	0.001	5
6	Language planning	0.045	0.154	0.005	0.030	6
7	International communication	0.045	0.001	0.001	0.001	7

#### Thematic content analysis.

In Table 2, the top 15 key terms for the seven topics were systematically ranked to further analyze their relevance.

**Table 2:** Terms and their relevance for theme analysis^a.^

	Topics	Term	Relevance	Term	Relevance	Term	Relevance
1	Language life	engineering	0.007	language life	0.006	languages	0.005
		network	0.007	poverty alleviation	0.005	grammar	0.005
		norms	0.006	methods	0.005	standard	0.004
		diversity	0.006	language resources	0.005	language protection	0.004
		media	0.006	advancement	0.005	professor	0.004
2	Language education	textbook	0.007	platform	0.005	school	0.005
		construction	0.006	university	0.005	French	0.004
		governance	0.006	education	0.005	consciousness	0.004
		policy	0.005	important	0.005	children	0.004
		disciplines	0.005	artificial intelligence	0.005	Chinese	0.004
3	Language resources	Chinese	0.008	language ability	0.006	center	0.005
		network	0.008	dialect	0.006	Chinese character	0.005
		center	0.007	director	0.006	collaborative	0.005
		language resources	0.007	demand	0.006	condition	0.005
		Mandarin	0.006	language and information division	0.005	vocabulary	0.005
4	Language protection	protection	0.007	native language	0.006	global	0.005
		language	0.006	person	0.006	terms	0.005
		communication	0.006	head	0.005	survey	0.005
		linguistic	0.006	institute	0.005	function	0.005
		theory	0.006	research	0.005	discussion	0.005
5	Language research	strategy	0.007	academic	0.005	English	0.005
		civilization	0.006	translation	0.005	center	0.005
		linguistic	0.006	development	0.005	cognitive	0.004
		monitoring	0.006	research	0.005	way	0.004
		college	0.005	subject	0.005	theory	0.004
6	Language planning	language policy	0.009	dialect	0.006	national languages	0.005
		think tank	0.008	linguistics	0.005	phenomenon	0.005
		planning	0.007	talent	0.005	analysis	0.005
		capacity	0.007	Mandarin	0.005	English	0.004
		era	0.006	community	0.005	forum	0.004
7	International communication	Belt and Road	0.010	national language commission	0.005	culture	0.005
		university	0.007	scientific research	0.005	human	0.005
		intelligence	0.007	informatization	0.005	America	0.005
		seminar	0.007	system	0.005	nation	0.005
		minority	0.006	scholar	0.005	world	0.005

^a^ The list of terms was derived from the LDA results using Gensim (K = 7) to automatically extract semantic topics.

Topic 1: Language life. Topic 1 featured terms such as engineering, network, norms, diversity, and media, highlighting the important role of digitization in promoting harmonious language development. Engineering and network exhibited the highest relevance of 0.007, associated with the language resources protection project initiated in 2015.

Topic 2: Language education. Topic 2 encompassed terms such as textbook, construction, governance, policy, and disciplines, emphasizing the collaborative efforts of government, education, and media entities. Textbook exhibited the highest relevance of 0.007, while construction and governance followed with a relevance of 0.006, highlighting the significance of language education, governance support and technological advances.

Topic 3: Language resources. Topic 3 reflected research priorities in the development and preservation of language resources. The relevance for Chinese and network were both 0.008, indicating Chinese language resources play an important role in the digital sphere.

Topic 4: Language protection. Topic 4 contained terms such as protection, language, communication, theory, and native Language, with protection garnering the highest relevance of 0.007. This topic delved into the objectives, current status, and significance of language protection, emphasizing the necessity of effective mechanisms to support the development and implementation of language protection initiatives.

Topic 5: Language research. Topic 5 covered terms like strategy, civilization, linguistic, monitoring, college, and academic, with strategy exhibiting the highest relevance of 0.007. This topic concentrated on different aspects of language research, including methodology, content, and institutional frameworks.

Topic 6: Language planning. Topic 6 included terms such as language policy, think tank, planning, capacity, and era, with language policy having the highest relevance of 0.009. The topic centered on the issues, content, progress, and organizational aspects of language planning, ranging from language policy to public linguistic life.

Topic 7: International communication. Topic 7 involved terms like Belt and Road, university, intelligence, seminar, and minority, with Belt and Road having the highest relevance of 0.010. This topic highlighted the significance of language in international communication, particularly emphasizing the Belt and Road initiative’s role in this context.

Overall, several key items were consistent with the word cloud, with particular attention to diversity, governance, grammar, Belt and Road, and strategies in the thematic analysis. This reinforced the emphasis on linguistic diversity, language planning and language education as priority areas.

### Theme intensity evolution analysis

**Fig 4:**
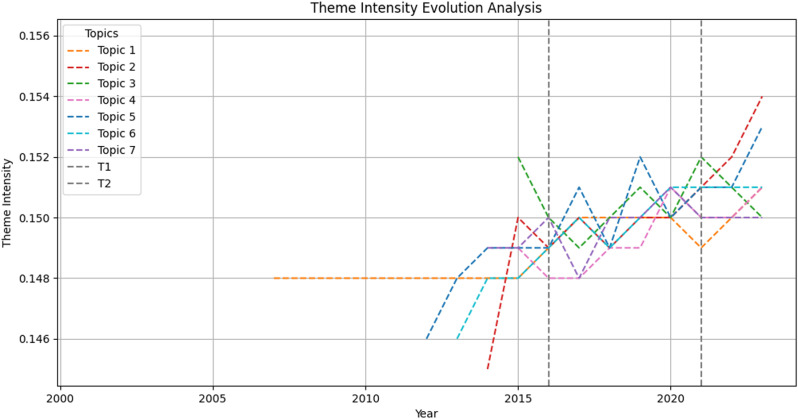
Theme intensity evolution analysis^a^. ^a^ T1 denotes the year 2016, while T2 signifies 2021. These specific time points were selected due to the first phase of the language resources protection project in China in 2016. Moreover, 2021 marks the subsequent phase in the development of the language resources protection project.

Fig 4 illustrated the trend of theme intensity for each topic, highlighting shifts in China’s language policy focus over different time periods. There were the following results: In general, the theme intensity of most topics has exhibited an upward trend since 2014, with significant fluctuations during T1-T2. Firstly, Topic 1 has shown an increasing trend since 2015, experiencing a decline to 0.149 in 2021 before rising again. This trend pattern corresponded with the release of the *13th Five-Year Plan for the Development of the National Language and Literature Program* and the *Implementation Plan for the Universalization of the State Common Language and Literature Project*.

Secondly, Topic 2 has shown continuous growth since 2014, reaching 0.154 in 2023, highlighting China’s emphasis on language education. The government launched the *National Universal Language and Script Popularization Project* in 2017, formulated the *Opinions on Comprehensively Strengthening Language and Literature Work in the New Era* in 2020 and proposed the *full implementation of education and teaching in the national common language and script* in 2022. These measures have significantly shaped language policy, enhancing both the accessibility and quality of language education. Topic 3, after experiencing a brief decline in 2015, peaked in 2019 and 2021 with a maximum value of 0.152. The first phase of the China Language Resources Protection Project concluded in 2019, and the launch of its second phase in 2021 reinforced efforts in language data mining and informed decision-making.

Thirdly, Topic 4 exhibited fluctuations before peaking at 0.151 in 2020, highlighting policymakers’ focus on language protection. Topic 5 fluctuated and grew, peaking at 0.151 in 2017 and 0.152 in 2019. Topic 6 showed a fluctuating upward trend, stabilizing at 0.151 after 2020. Finally, Topic 7 experienced fluctuating growth, peaking at 0.151 in 2020 after a brief decline in 2017. In 2020, the Ministry of Education established the *Center for Sino-foreign Language Exchange and Cooperation* and the *China International Chinese Language Education Foundation*. These initiatives have promoted cultural exchanges and enhanced international understanding.

### Prediction of topic evolutionary trends

#### Time series difference processing and test.

The ADF test is widely employed as a prevalent unit root test for evaluating the stationarity of time series data [85]. Table 3 indicated that the p-value for all topics, except Topic 5, exceeded 0.05, indicating non-stationarity. However, the p-value dropped below 0.05 after differencing, suggesting that stationarity was achieved. Topic 5 passed the ADF test, indicating stationarity at the 0.1% significance level, while the remaining topics exhibited stationarity in their time series after differencing.

**Table 3:** Time series ADF test results^a.^

Topics	Difference	t-statistic	Test critical values			p-value
			1% level	5% level	10% level	
Topic 1	d = 0	-1.943	-4.380	-3.600	-3.240	0.632
	d = 2	-4.609	-4.380	-3.600	-3.240	0.001***
Topic 2	d = 0	-4.277	-4.380	-3.600	-3.240	0.003*
	d = 1	-8.203	-4.380	-3.600	-3.240	0.001***
Topic 3	d = 0	-3.093	-4.380	-3.600	-3.240	0.108
	d = 2	-4.486	-4.380	-3.600	-3.240	0.002**
Topic 4	d = 0	-3.279	-4.380	-3.600	-3.240	0.070
	d = 2	-3.490	-4.380	-3.600	-3.240	0.041*
Topic 5	d = 0	-5.632	-4.380	-3.600	-3.240	0.001***
Topic 6	d = 0	-2.575	-4.380	-3.600	-3.240	0.292
	d = 3	-3.451	-4.380	-3.600	-3.240	0.045*
Topic 7	d = 0	-3.204	-4.380	-3.600	-3.240	0.084
	d = 1	-4.563	-4.380	-3.600	-3.240	0.001***

^a^ *p < 0.05, **p < 0.01, ***p < 0.001.

#### Parameter estimation and model validation.

We performed the Ljung-Box test on the residual series to assess the robustness of the fitting and predictive abilities for each topic in Table 4. Firstly, we selected the parameter with the $\frac{\left(AIC+BIC\right)\;}{2}$ minimum to establish optimal models for Topic 1–7. Secondly, the Ljung-Box test results for all topics exhibited p ＞ 0.1, indicating that there was no significant autocorrelation. Specifically, Topic 1 addressed trend effects through second-order differencing. The optimal model, ARIMA(2,2,0), was identified with parameters p = 2 and q = 0, achieving the $\frac{\left(AIC+BIC\right)\;}{2}$ minimum (AIC = -133.565, BIC = -131.305). The Ljung-Box Q(6) test yielded a p-value of 0.314, confirming the absence of significant autocorrelation and validating the model’s suitability.

**Table 4:** ARIMA model parameter estimation and Ljung-Box test results.

Topics	ARIMA(p,d,q)	p-value	AIC^a^	BIC^a^	Ljung-Box Q(6)^b^
Topic 1	ARIMA(2,2,0)	0.001***	-133.565	-131.305	0.314
Topic 2	ARIMA(1,1,0)	0.001***	-72.557	-72.318	0.501
Topic 3	ARIMA(1,1,0)	0.050*	-49.500	-50.124	0.728
Topic 4	ARIMA(2,1,0)	0.004**	-52.253	-53.086	0.581
Topic 5	ARIMA(1,0,0)	0.006**	-118.921	-117.226	0.929
Topic 6	ARIMA(1,1,0)	0.030*	-37.918	-39.090	0.513
Topic 7	ARIMA(1,1,0)	0.010**	-87.031	-86.439	0.151

^a^ The model that exhibited the most optimal fit and featured all model parameters that were statistically significant was chosen by employing the criteria of $\frac{\left(AIC+BIC\right)\;}{2}$ minimum.

^b^ The Ljung-Box test with p > 0.05 did not provide sufficient evidence to reject the null hypothesis that there is no autocorrelation.

#### Results of topic evolutionary trends prediction.

**Fig 5:**
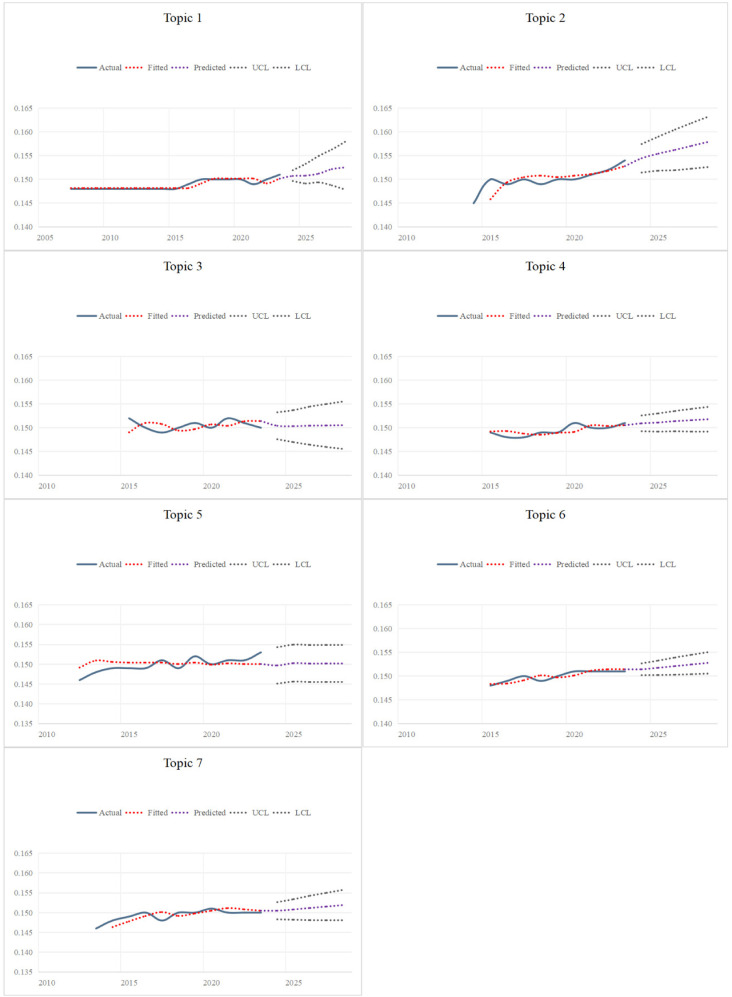
Results of the trend prediction of China’s language policy^a^. ^a^ The UCL and LCL confidence levels are obtained by extrapolating the sample data, where UCL denotes the high limit of the confidence interval and LCL denotes the low limit of the confidence interval.

To understand the dynamics of China’s language policy, we analyzed the theme intensity within the raw time-series data from 2001 to 2023 employing an ARIMA model, then we predicted the trend of seven topics from 2024 to 2028, as shown in Fig 5.

Regarding the actual values, Topics 1, 2, 4, 5, 6 and 7 exhibited an increasing trend from 2001 to 2023. Topic 2 exhibited the highest increase, rising from 0.145 to 0.154, with an increase of 0.009. While Topic 3 showed a fluctuating trend, peaking at 0.152 in 2022 before gradually decreasing. In terms of the fitted values, Topics 1, 2, 3, 4, 6 and 7 exhibited an overall upward fluctuation from 2001 to 2023. Topic 4 peaked at 0.151 in 2022 before stabilizing, while Topic 5 remained relatively stable at 0.150. Fig 5 illustrated the similarity between the fitted series and the actual series, verifying the overall effectiveness of the model in capturing the time series of theme intensity.

In terms of predicted values, Topics 1, 2, 4, 5, 6 and 7 exhibited positive trends between 2024 and 2028, with Topic 2 showing the fastest growth. Meanwhile, Topic 3 was expected to gradually stabilize following a brief decline from its peak of 0.152 in 2023. In summary, the predicted values for all seven topics fell within the 95% confidence interval, indicating the high accuracy of the model in forecasting the data.

#### Evaluation of autoregressive integrated moving average model efficacy.

To assess the efficacy of the ARIMA model, RMSE and MAPE were used as key metrics for evaluating model performance [86,87], as detailed in Table 5. The findings indicated that the RMSE values approached zero across all seven topics, while the MAPE values consistently remained below 5%. These results highlighted the model’s strong predictive accuracy, affirming the method’s effectiveness.

**Table 5:** ARIMA model predictive assessment.

Predictive model	Topic 1 ARIMA(2,2,0)	Topic 2 ARIMA(1,1,0)	Topic 3 ARIMA(1,1,0)	Topic 4 ARIMA(2,1,0)	Topic 5 ARIMA(1,0,0)	Topic 6 ARIMA(1,1,0)	Topic 7 ARIMA(1,1,0)
RMSE	0.003	0.004	0.004	0.002	0.005	0.002	0.003
MAPE(%)	1.667	2.686	2.630	1.418	3.104	1.169	2.006

## Discussion

Language policy serves as a crucial instrument for governments to regulate and guide language development, thereby attracting significant academic interest [88–90]. This study employs the LDA unsupervised machine learning method and the ARIMA model with high predictive accuracy to analyze 1,420 China’s language policy texts. The analysis identifies seven topics and forecasts their evolutionary trends over the next five years, aiming to enhance the effectiveness of language policy.

LDA is an effective large-scale thematic model for textual analysis used to assess the topics and intensities embedded in policy texts [91]. Topic 1 has the highest prevalence index of 0.318 and a high expectation value of 0.153, indicating significant social concern about language use and development. The thematic content analysis emphasizes the importance of language texts, knowledge, and emerging technologies in shaping language life. The trend of theme intensity evolution has shown a continuous increase since 2015, with a brief decline in 2021, followed by a renewed rise. In 2022, China achieved an 80.72% penetration rate of Mandarin, showcasing advancements in promoting the national language [92]. As China works towards standardizing its language, its linguistic values are also diversifying, resulting in a rich and colorful language life where various languages and dialects coexist, reflecting the characteristics of the times.

Topic 2 has the second-highest popularity index of 0.227 and the highest expectation value of 0.154, highlighting its significance in language policy. The thematic content analysis reveals the significant role that textbooks and discipline building in enhancing the quality of language education. Scientific language policy and management serve as powerful tools for promoting educational reform and enhancing cultural literacy [93]. The theme intensity has shown continuous growth since 2014, reaching 0.154 in 2023, indicating China’s strong emphasis on language education. In 2020, the proportion of the literate population using standardized Chinese characters exceeded 95.00%, and the illiteracy rate dropped to 2.67% [94]. Despite significant progress, challenges persist, such as the limited variety of teaching materials and the insufficient quality of teaching staff [95,96]. Therefore, it is imperative to adjust and enhance language education policies, implement reforms to optimize academic disciplines development.

The high popularity index of 0.182 for Topic 3 underscores the importance of the Chinese on digital platforms, as well as the crucial role of language proficiency, dialects, and Mandarin in fostering sustainable language resource development. The theme intensity of language resources fluctuated in stages, reflecting gradual adjustments in China’s language resource protection initiatives. At present, China has established the world’s largest language resource database, encompassing over 120 languages and dialects [97]. While the development of language resources can yield economic benefits, it also faces challenges, such as balancing the relationship between common languages and dialects and ensuring the protection of vulnerable language groups.

Topic 4 emphasizes language preservation through a comprehensive policy strategy aimed at sustaining linguistic vitality. The intensity peaked at 0.151 in 2020. In recent years, the government has protected languages and scripts by enacting laws, setting up dedicated agencies, and executing projects to protect language resources [98,99]. Despite significant progress in language preservation, challenges persist, including the great variety of languages and the rapid rate of endangerment. Exploring innovative technologies and methods is crucial to address these issues.

Topic 5, with an expectation value of 0.153, focuses on the methods and content of language research, highlighting the importance of language strategies and monitoring to understand the state of languages and support language policy development. The trend of theme intensity showed fluctuating growth, indicating sustained interest in language research. However, current language research tends to favor analysis, neglecting the study of real discourse and language function [100,101]. Effectively addressing the complexities of language research demands a multidisciplinary approach.

Topic 6, with the highest expectation value of 0.154, explores the benefits of intelligent language construction through AI and big data. The theme intensity has stabilized since 2020. The Chinese government has strengthened the management system for language and writing recently. More than 80 laws and regulations, such as the *Law on the State Common Language and Writing System* and *administrative rules concerning the Putonghua Shuiping Ceshi*, have been established [98,102]. These efforts in language planning have provided significant support and guarantees for economic, social, and linguistic development.

Topic 7 highlights the importance of language in international exchange, particularly the strategic role of the Belt and Road Initiative. The theme intensity has fluctuated and grown, peaking at 0.151 in 2020. By 2019, the number of Chinese language learners worldwide had surpassed 100 million, with more than 500 Confucius Institutes established globally [103]. This underscores the need to integrate economic growth with strengthened bilateral and multilateral language and cultural exchanges to foster international understanding and cooperation.

The ARIMA model was used to predict the trends of seven topics over the next five years. Fig 5 shows that, with the exception of language resources (Topic 3), the predicted values for the other six topics will exhibit an upward trend from 2024 to 2028. Notably, the predicted value for language education (Topic 2) is expected to show the most significant growth. In contrast, Topic 3 will stabilize after a brief decline, while the predicted value for language research (Topic 5) will remain steady at 0.150. Language education has consistently attracted considerable attention, and projections suggest this trend will persist. This underscores the significance of language education in language policy, particularly the role of foreign language education in promoting language policy implementation and international communication. Language resources peaked in 2021, attributed to the initial success of the language protection project, which heightened government and societal attention to the development and utilization of language resources. However, model projections indicate that language resources will gradually level off after a brief decline in 2023. This trend is attributed to rapid economic growth and urbanization, which pose risks of language endangerment or disappearance, especially in economically developed cities and regions. The relatively stable predicted value for language research indicates sustained interest in this area. Language research primarily focuses on topics such as language governance, language translation, and research methodologies, reflecting the capacity of language policy to provide technical and strategic support.

Based on the obtained discussion, this study proposes the following optimization paths.

Firstly, establish a collaborative language education system involving the government, schools, and society. The government should integrate language education into its top-level design and provide public services. Schools must advance inclusive language education, bridging urban-rural gaps. Society should organize regular language education activities using existing resources. This cooperation is essential for developing balanced and sustainable language policies.

Secondly, robust language research is crucial for fostering a harmonious linguistic life and refining language planning. Advancements in language research can support linguistic diversity and enhance language governance through initiatives such as promoting common language, surveying dialect and protecting endangered languages.

Thirdly, strengthening regional connectivity is essential for optimizing language resource management. Given China’s regional economic variations and different urban backgrounds, it is imperative to develop region-specific language industries that fully utilize local language resources.

## Conclusions and limitations

This study investigated 1,420 policy texts from official government websites, extracting seven topics using LDA and ARIMA models. The study drew the following conclusions: (1) Language life, language education, and language resources hold a prominent place in China’s language policy, indicating the country’s commitment to building a harmonious linguistic environment, addressing educational inequality, and protecting language resources. (2) Since 2014, most topics have shown an upward trend, especially language education and language research, reflecting significant efforts by the Chinese government to achieve economic and social benefits. (3) Prediction trends indicate that, except for language resources, the predicted values of the other six topics will show a positive trend from 2024 to 2028, with language education demonstrating particularly strong growth. Based on these findings, we propose policy recommendations such as deepening language research, developing a multilingual education system, and optimizing language resource management.

However, there are still some limitations: (1) Topic modeling limitations. Thematic extraction results are presented in an abstract or data-oriented manner, requiring integration with relevant contextual backgrounds for full comprehension. However, this focus on identifying innovative research points may result in biased conclusions, potentially neglecting broader research fields. (2) ARIMA model limitations. The ARIMA model, despite its simplicity and interpretability, displays sensitivity to data outliers and does not account for noise, the characterization of which remains unclear. (3) Data limitations. The primary dataset comprised official policies and news, which could introduce validation bias. Addressing such bias falls beyond the scope of this study.
